# Cross-sectional multicentre study on the cohort of all the French junior lecturers in general practice

**DOI:** 10.1080/13814788.2017.1422176

**Published:** 2018-01-24

**Authors:** Marie Barais, Catherine Laporte, Matthieu Schuers, Olivier Saint-Lary, Paul Frappé, Clarisse Dibao-Dina, David Darmon, Tiphanie Bouchez, Julien Gelly

**Affiliations:** ^a^ EA SPURBO, Department of General Practice, Université de Bretagne Occidentale Brest France; ^b^ Department of General Medicine, Faculty of Medicine, UFR Medecine Clermont-Ferrand France; ^c^ Department of General Practice, Department of Biomedical Informatics, Rouen University, Rouen University Hospital, LITIS EA 4108, Normandie University Rouen France; ^d^ Faculty of Health Sciences Simone Veil, Department of Family Medicine, Université Versailles Saint-Quentin en Yvelines Montigny le Bretonneux France; ^e^ CESP, INSERM (U1018), Team 1: “Health Services Research”, Université Versailles Saint-Quentin en Yvelines Versailles France; ^f^ Department of General Practice, University of Saint-Etienne Saint-Etienne France; ^g^ Department of General Practice, François Rabelais Tours University Tours France; ^h^ Department of Education and Research in General Practice, Nice Sophia-Antipolis University Nice France; ^i^ Department of General Practice, Paris Diderot University, Inserm, IAME, UMR 1137, Paris Diderot University Paris France

**Keywords:** General practice, research, teaching, delivery of healthcare

## Abstract

**Background:** General practice became an academic discipline quite recently in many western countries. In France, junior lecturer work is specified in a three-part mandate: medical work in general practice, teaching in the university, and research. Since 2007, 130 junior lecturers have been appointed in general practice. The aim of the creation of junior lecturer status was to align general practice with other specialties and to develop research and education in primary care.

**Objectives:** To describe the healthcare, teaching and research undertaken by junior lecturers in general practice, practising in October 2014.

**Methods:** A cross-sectional multicentre study using an online self-administered questionnaire on the cohort composed of all the junior lecturers in general practice with open questions and the qualitative analysis of written verbatim accounts.

**Results:** Of the 95 junior lecturers practising at the date of the study, 75 (79%) responded; average age 32 years; gender ratio (F/M) 2.4:1. They spent five, two and three half-days per week respectively in healthcare, teaching and research. The healthcare activity was predominantly carried out in the community (73%). Thirty-nine per cent worked as part of a multi-professional team taking on 50 consultations per week. Most of the educational work involved lecturing and mentoring students specializing in general practice (median 86 hours per year). Research output increased during the fellowship. Research topics were varied and relevant to the disciplinary field.

**Conclusion:** During the fellowship, the balancing, and even the reinforcement, of healthcare and research contributions were accompanied by a significant investment in educational provision.

KEY MESSAGES

- In contrast to the previous generation of GPs, French junior lecturers are young (32 years old), and predominantly women (gender ratio 2.4:1).
- Since the creation of the junior lecturer position, publications in peer-reviewed journals by French teams from general practice have increased.

## Introduction

General practice is now an academic discipline in most western countries. However, the route to becoming an academic general practitioner (GP) differs by country. Academic clinical fellowships for GP specialist trainees, academic in-practice fellowships for fully trained GPs, clinical lectureships and senior clinical lectureships leading to a professorial post, are different routes for career progression [1].

In France, general practice has existed as an academic discipline since 2008. This recent academic development in general practice explains the low rate of publication by French general practice departments. When graduates have chosen to become GPs, they have to follow three years of six-month internships. Two internships take place at GP surgeries where the trainees learn to be autonomous. The volunteer GPs involved are known as ‘GP trainers’ after specific training. After graduation, trainees can choose to become ‘junior lecturers.’ They have to apply for this position after meeting recruitment criteria defined by the department of general practice. Their work is specified in a three-part mandate: medical activity in a general practice surgery, teaching in the university, and research [2]. They are bound by a contract with the university, from which they receive a salary. We chose the term ‘junior lecturer’ to describe this type of French academic status. The first junior lecturers in general practice were appointed in France in 2007. Junior lecturer status has existed in other medical and surgical specialties in university hospitals in France since 1958. The aim behind the creation of the junior lecturer position was to align general practice with other specialties and to develop research and education in primary care.

The maximum duration of the junior lecturers’ mission is four years. After fulfilling their mandate, they can choose the academic path and become senior lecturers and then full professors.

On 1 January 2015, 130 junior lecturers were in place in the 37 French university departments of general practice. They worked with 37 full professors, 72 associate professors, 13 senior lecturers, 128 associate lecturers and 7863 GP trainers. There were 14 207 students registered as general practice trainees. Among academic staff in general practice, the teacher–student ratio is 1:97, whereas this ratio of teaching staff to students is ten times higher in the other medical disciplines [3,4].

There is no data on the daily workload of junior lecturers following the application of this extension. This study aimed to describe the working profile (healthcare, teaching and research work) of the cohort of French junior lecturers in general practice seven years after the creation of academic status. By describing this working profile, we wanted to illustrate how the development of junior lecturer status established the academic position of French primary care.

## Methods

### Study design

We have undertaken a cross-sectional multicentre study, using an online self-administered questionnaire, on the cohort composed of all the junior lecturers in general practice and with a qualitative analysis of written verbatim accounts to open questions at the end of the questionnaire.

### Setting

The survey was conducted from 16 September to 30 October 2014. Each university was a centre and there were several junior lecturers in each centre.

### Participants

Using an exhaustive email list, we invited all French junior lecturers and associate lecturers to take part.

### Variables

The questionnaire was made up of 220 questions divided into ten parts. The answers were divided into single answers, multiple-choice answers for quantitative questions and free text for qualitative questions.

The quantitative part of the questionnaire had been developed from a previous study undertaken in 2011. Qualitative data were derived from a previous qualitative study, which aimed to explore the feelings of the junior lecturers about their mission.

The length of time required to fill in the questionnaire was estimated at 45 min. LimeSurvey^®^ v 2.05 was used for the study. Reminders were sent to the non-respondents.

### Study size

The cohort of all the French junior lecturers was at our disposal. We did not estimate the size of the sample but submitted the questionnaire to all of them.

### Quantitative variables

Quantitative data were collected concerning healthcare, educational, and research work which focused on organization, content, training, job satisfaction, and prospects.

### Statistical methods

Numeric variables were described with median (minimum-maximum) and categorical variables with sample size (proportion). Owing to the small size of the population, non-parametric tests were used to compare results between the different years of seniority: Kruskal–Wallis test for numeric variables and Fisher’s exact test for the categorical variables. The respondents were divided into categories related to their seniority within their mandate: first year, second year, third year, fourth year, fifth year and beyond.

### Qualitative data

A thematic analysis was conducted using the technique of constant comparison, which originated in grounded theory [5]. Open coding was done by two researchers (CL and TB), working independently, without any framework for the written data. Open codes were shared and any discrepancies were discussed with one member of the research team (CDD) until a consensus was reached. Then an axial coding framework was developed using an iterative process of constant comparison. The axial coding involved linking categories found within the open coding. The same procedure of working independently before sharing the results was applied. The study was registered with the Local Commission on Information Technology and Liberties (CIL) of the University of Auvergne.

## Results

### Participants

Ninety-five junior lecturers in general practice were practising at the date of the study in France. Seventy-five (79%) responded. Their median age was 32 years (from 25 to 40). Female/male ratio was 2.4:1. Forty-five (60%) were parents with a median of two children.

### Quantitative data

*Description of the healthcare work.* They spent five, two and three half-days per week respectively in healthcare, teaching and research. The healthcare work was predominantly (73%) carried out in the community. Thirty-nine per cent worked in a multi-professional team, taking on 50 consultations per week on average. The number of patients reporting them as their main doctor increased during the fellowship, from 35 in the first year to 225 in the fifth year and beyond. The median duration of the consultation was 20 min (from 15–30 min). Forty-three junior lecturers (57%) were part of an on-call service. Table 1 describes healthcare work according to level (by yearly progression).

**Table 1.** Details of healthcare work according to level (by yearly progression) on 1 October 2014.

	First year (*n* = 20)	Second year (*n* = 29)	Third or fourth year (*n* = 20)	Fifth year and more (*n* = 20)	*p*
Practising					
Number of half-days per week	5 (4–8)	6 (0–8)	5 (2–9)	5 (1–7)	0.34
Number of patients on the list	35 (0–700)	200 (0–550)	183 (0–740)	225 (30–600)	0.02
Number of consultations per week	50 (0–85)	55 (30–90)	49 (25–90)	58 (40–75)	0.47
On-call service	12 (60%)	14 (48%)	9 (45%)	8 (40%)	0.68

*Description of the educational activity.* Most of the educational work related to students specializing in general practice. Lectures and small-group tuition represented a median of 86 h per year (63–150 h). They were also involved in a continuous training programme as organizers, facilitators or experts. Twenty-five (33%) junior lecturers were tutors in their own practice. Table 2 describes the breakdown of educational tasks by level of yearly progression.

**Table 2.** Breakdown of educational tasks according to level (by yearly progression) on 1 October 2014.

Teaching					
Number of half-days per week	2 (1–6)	2 (1–6)	2 (1–5)	1 (0–3)	0.10
Certified degree in medical education	12 (60%)	28 (97%)	15 (75%)	12 (60%)	0.003
University degree medical education	7 (35%)	11 (38%)	10 (50%)	10 (50%)	0.69
Number of hours teaching per year	79 (64–88)	112 (63–150)	100 (74–120)	75 (75–75)	0.57
Large groups	48 (15–72)	49 (6–100)	20 (4–83)	4 (4–4)	0.13
Small groups	30 (0–170)	59 (15–106)	80 (14–116)	71 (71–71)	0.26
Number of hours teaching per year per degree	0 (0–8)	0 (0–27)	0 (0–11)	24 (24–24)	0.43
From first to third year	6 (0–34)	11 (0–100)	16 (4–30)	8 (6–14)	0.79
From fourth to sixth year	56 (14–106)	53 (14–85)	68 (17–106)	45 (30–90)	0.37
Vocational trainee in primary care					
Classroom practitioner	8 (40%)	12 (41%)	6 (30%)	8 (40%)	0.87
Pre-graduate medical student	5 (25%)	12 (41%)	3 (15%)	7 (35%)	0.23
First ambulatory internship	3 (15%)	1 (3%)	3 (15%)	3 (15%)	0.36
Second ambulatory internship	1 (5%)	1 (3%)	1 (5%)	4 (20%)	0.22
Number of half-days per week	2 (1–9)	3 (1–9)	4 (2–7)	4 (2–8)	0.10
Pre-graduate medical student	2 (1–6)	3 (1–5)	5 (3–6)	2 (2–6)	0.17
Trainee first level	2 (2–4)	4 (4–4)	2 (2–6)	3 (1–3)	0.72
Trainee second level	3 (3–3)	2 (2–2)	1 (1–1)	3 (2–4)	0.38
Tutor	9 (45%)	17 (59%)	11 (55%)	6 (30%)	0.23
Number of tutored trainees	3 (1–8)	6 (1–16)	7 (5–11)	6 (3–12)	0.24
Service on an exam board	12 (60%)	22 (76%)	12 (60%)	10 (50%)	0.30
Defence of dissertation for graduation	1 (0–4)	4 (1–12)	4 (0–11)	10 (0–80)	0.001
Defence of dissertation for degree in medicine	4 (0–80)	1 (0–6)	4 (0–11)	8 (4–20)	≤0.001
Number of times involved in national health medical continuing education	8 (40%)	11 (38%)	7 (35%)	11 (55%)	0.59
As team leader	6 (30%)	9 (31%)	7 (35%)	9 (45%)	0.76
As expert	2 (10%)	3 (10%)	–	7 (35%)	0.01
As organizer	4 (20%)	2 (7%)	2 (10%)	4 (20%)	0.41
Number of times involved in continuing education	2 (1–4)	3 (1–4)	3 (2–9)	4 (1–10)	0.07
As team leader	1 (1–3)	2 (1–4)	3 (2–9)	2 (1–4)	0.17
As expert	1 (1–1)	1 (0–1)	–	3 (2–8)	0.06
As organizer	1 (1–1)	1 (1–1)	1 (1–1)	1 (1–2)	0.57

*Description of the research tasks.* Research output increased during the fellowship. Most of the junior lecturers had training in research and an ongoing project (45% in a registered group). Thirty (40%) junior lecturers had published one or more articles in a journal indexed in PubMed (irrespective of author ranking), from five in the first year to 11 in the fifth year and beyond. Thirty junior lecturers had published one or more articles in a journal not indexed in PubMed (irrespective of author ranking) from five in the first year to 10 in the fifth year and beyond. Oral communications and scientific posters in international conferences increased in line with seniority from zero in the first year to one in the fifth year and beyond (*p* <0.001 and *p* <0.001, respectively). Thirty-two (43%) were tutors for the Master’s thesis to become a Doctor of Medicine. Table 3 describes the breakdown of research tasks by level of yearly progression.

**Table 3.** Breakdown of research tasks according to level (by yearly progression) on 1 October 2014.

Research					
Number of half-days per week	3 (1–6)	2 (1–5)	2 (1–6)	4 (2–6)	0.03
Training in research	16 (80%)	19 (66%)	15 (75%)	12 (60%)	0.51
PhD	–	–	2 (10%)	2 (10%)	0.16
(in progress)	3 (15%)	4 (14%)	–	6 (33%)	0.04
Master 2	9 (45%)	10 (34%)	11 (55%)	10 (50%)	0.54
(in progress)	1 (9%)	4 (21%)	1 (11%)	–	0.54
Master 1	7 (35%)	12 (41%)	6 (30%)	8 (40%)	0.88
(in progress)	–	–	–	–	–
University degree	–	2 (7%)	1 (5%)	1 (5%)	0.90
National society (CNGE, SFMG, SFTG, etc.)	1 (5%)	4 (14%)	3 (15%)	3 (15%)	0.77
Summer or autumn school	6 (30%)	9 (31%)	7 (35%)	3 (15%)	0.50
Research course	–	2 (7%)	1 (5%)	2 (10%)	0.76
Other	2 (10%)	3 (10%)	3 (15%)	3 (15%)	0.92
Training in software	16 (80%)	15 (52%)	6 (30%)	10 (50%)	0.13
Bibliography software	11 (55%)	13 (45%)	9 (45%)	8 (40%)	0.83
Quantitative research software	9 (45%)	11 (38%)	13 (65%)	7 (35%)	0.23
Qualitative research software	6 (30%)	8 (28%)	9 (45%)	6 (30%)	0.67
Questionnaire software	2 (10%)	7 (24%)	2 (10%)	2 (10%)	0.43
Registered research unit	11 (55%)	9 (31%)	7 (35%)	10 (50%)	0.30
Publication non-indexed in PubMed	5 (25%)	8 (28%)	7 (35%)	10 (50%)	0.33
Number of publications as first author	0 (0–4)	0 (0–11)	0 (0–3)	0 (0–3)	0.15
Number of publications, other rank	0 (0–2)	0 (0–5)	0 (0–3)	1 (0–5)	0.009
Publication indexed in PubMed	5 (25%)	7 (24%)	7 (35%)	11 (55%)	0.13
Number of publications as first author	0 (0–8)	0 (0–2)	0 (0–7)	0 (0–5)	0.13
Number of publications, other rank	0 (0–3)	0 (0–3)	0 (0–3)	1 (0–3)	0.03
Number of oral communications	10 (50%)	18 (62%)	10 (50%)	11 (55%)	0.83
National conferences	0 (0–10)	1 (0–4)	0 (0–11)	4 (0–34)	0.16
International conferences	0 (0–2)	0 (0–1)	0 (0–6)	1 (0–6)	0.04
Number of posters	7 (35%)	16 (55%)	6 (30%)	10 (50%)	0.28
National conferences	0 (0–6)	1 (0–4)	0 (0–4)	0 (0–11)	0.37
International conferences	0 (0–3)	0 (0–2)	0 (0–12)	0 (0–6)	0.01
Number of theses supervised	2 (0–21)	0 (0–2)	1 (0–7)	5 (2–21)	≤0.001
(in progress)	3 (1–10)	3 (1–10)	3 (2–8)	4 (1–8)	0.86
Number of theses co-supervised	1 (0–1)	2 (0–2)	1 (1–3)	1 (1–3)	0.15
(in progress)	1 (0–2)	2 (0–3)	1 (0–2)	3 (1–4)	0.11
Number of methodological studies supported	2 (0–15)	2 (0–15)	4 (2–10)	5 (2–20)	0.11
(in progress)	5 (1–15)	2 (0–15)	3 (0–10)	5 (4–15)	0.42

### Qualitative data

The three roles and types of workload were seen as difficult to reconcile.

While the educational work was considered of less value for an academic career, it brought with it more immediate feelings of gratification, from contact with the students, than the other types of work.

The lack of resources was the main characteristic in the research area: funds (for studies or faculty positions), human resources (other teacher-researchers in other departments, methodologists, statisticians, translators, mentors for the PhD), organizational resources (research unit, institutional partners, dedicated space, contact with other specialties), individual resources (professional skills, education). The lack of time to publish was frequently cited. The time dedicated to the practice and education was incompressible, in contrast to the research work. Involvement in a dynamic research team, with a helpful mentor and with the support of the department, encouraged output. Choosing one’s research theme was seen as motivating.

## Discussion

### Main findings

The experience of being a French junior lecturer correlated with an upgrading of the curriculum in the fields of healthcare, education and research. The junior lecturers who had been practising for four years were more involved in the area of healthcare practice and had more patients in their care. They had all obtained certified training in teaching in their fifth year. Their educational responsibilities in the department were greater, with a higher involvement in the academic education sessions. One third were also tutors in their practice. The older junior lecturers were more involved in research. Half of them had published an article in an indexed journal.

### Strengths and limitations

As far as we know, this study was the first to describe the roles and workload of French junior lecturers in the three dimensions of healthcare, education, and research. The total of 95 junior lecturers recruited throughout France may be considered limited in number but they made up the entire sample available. The response rate of 79% reveals a high level of involvement on the part of the junior lecturers in their willingness to share information related to their mandate.

The declarative form of the study is one of the major limitations. The data related to the number of publications were not checked against bibliographic databases. The data related to healthcare practice were not checked with the national social security individual annual report. The junior lecturers were not asked whether they had served on university boards or in institutions, such as the French National Authority for Health (Haute Autorité de Santé), or about their experience as reviewers. These points should be added in the follow-up to this cohort. We wanted to collect data on the junior lecturers’ contribution to education and research. Analysing their experiences as junior lecturers through semi-structured interviews, knowing the added value of this curriculum in the daily life of these GPs/teachers and researchers could be the objective of a second study.

Our data are three years old which could be considered a limitation. However, the regulatory framework has not changed and there seems to have been little change since that time in the dynamics within the three domains of research, practice, and education.

### Comparison with existing literature

Cronholm et al. described a Family Medicine Research Fellowship from the University of Pennsylvania, which was identical to the French junior lecturer status [6]. The division of the three-part mandate was similar to the French one. The outcomes of the fellowships were successful with 15 fellows and 114 articles being peer reviewed between 1997 and 2009. In the authors’ view, the goals had been achieved, in terms of providing competent GPs, as well as trained researchers in general practice who had become leaders in the discipline. ‘Family practice’ has existed as a discipline since 1969 in the USA [7], and one hypothesis explaining these successful results could be the experienced leadership. The competence of the mentors in research fields, as well as in the publication process, grant applications and the quality of their relationships with their peers, were the key to the success of the programme [6]. For the participants of our study, the research output was the slowest aspect of the role to develop. Designing and completing studies, setting up partnerships and publishing articles require time. Time spent conducting research was associated with greater productivity in Steiner’s study: it could be stating the obvious, but having sufficient time to do research is at the heart of the matter [8,9]. Another difference lies in the absence of a dedicated curriculum in research training in the French general practice department. Creating such a course may help the French junior lecturers to develop research activities earlier in their careers. Curtis et al. compared the training programmes and career paths of family physicians included in a national research programme with those of internists and paediatricians [10]. Whereas one-third of paediatricians and internists published one or more articles per year, only one-tenth of family physicians were published. One explanation for such a difference was that family physicians spent far more time on clinical work with patients, and less on doing research.

Alongside the time consideration, the culture of research needs to be developed in general practice. Bolon et al. highlighted the lack of involvement in research as the ‘major hurdle to teaching, mentoring, and involving fellows in research’ [11]. The weaker the research culture, the lower the level of research training undertaken in fellowships [11]. As a young academic discipline, French general practice is seriously deficient in this cultural area.

An Australian team describes the successful strategy of the government to build research capacity [12]. A national budget was provided in bursaries, grants, and the provision of research fellows to develop a specific research area in primary healthcare [12].

French researchers in general practice have to apply for a national medical grant, without any distinction being made between primary care and other disciplines. A dedicated budget for research in general practice has not yet been developed in France.

### Implications for research

Research themes were varied and they broadly covered the specific area of primary care. This made it possible to explore all the topics recommended by the research agenda, defined by the European General Practice Research Network [13]. This variety is also beneficial given the opportunities it creates to practise alongside academic GP leaders trained in research and education.

Mixing clinical practice, education and research in the same programme was a challenge for French junior researchers in 2007. The outcomes obtained in the areas of education and research have to be interpreted in the context of a new academic discipline without specific grants or a research culture.

The scientific relevance of research in general practice is beyond doubt. Hobbs et al. recognized the link between general practice and its academic discipline [14]. This link is the key to the results of studies conducted in primary care, the adaptation of clinical guidelines and better disease management. In addition, the interaction between professionals, and the attraction of the specialty for future GPs, would reinforce the GP workforce [13,14]. The lack of academic future was considered a constraint for students choosing general practice as a profession [15]. The academic opportunities created in a general practice department, which has its academic status must also be stressed.

## Conclusion

The French junior lecturers described in this study were young (32 years old), mainly women (gender ratio 2.4:1). Since the junior lecturer role was created, publications in peer review journals have increased for the French general practice teams. This study illuminates the building of the academic discipline in general practice in France by drawing attention to the outcome of the research and educational contributions made by the youngest members of the discipline.

## Ethics

The study was registered and approved by the Local Commission on Information Technology and Liberties (CIL) of the Auvergne University.
